# Supplementary material for: IGF2BP1—An Oncofetal RNA-Binding Protein Fuels Tumor Virus Propagation
Source: Viruses. 2023 Jun 24;15(7):1431. doi: 10.3390/v15071431 (PMC10385356; doi:10.3390/v15071431)
Supplement: Supplementary file 1 [file viruses-15-01431-s001.zip › viruses-2433801-supplementary.pdf]

Supplementary Materials: IGF2BP1 - an oncofetal RNA-binding protein fuels tumor virus propagation

Markus Glaß and Stefan Hüttelmaier

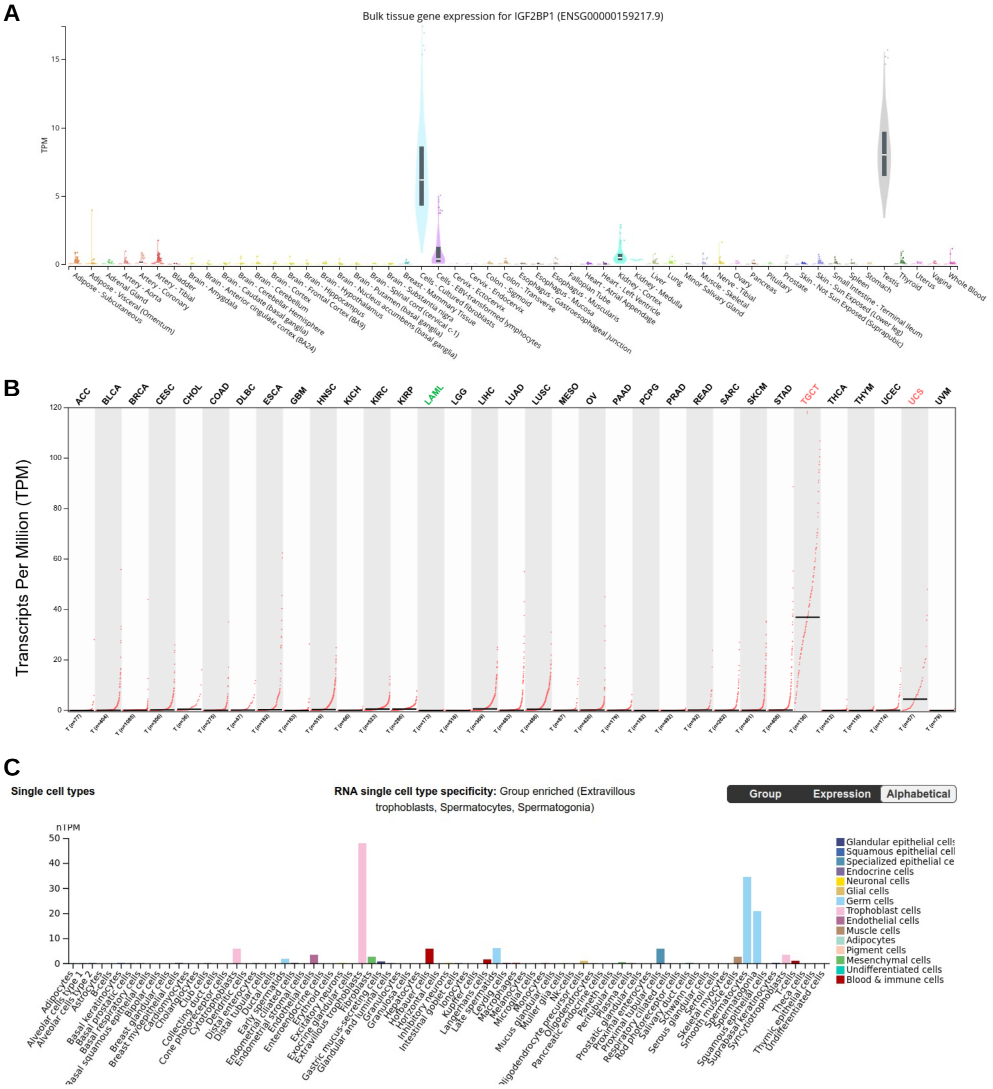

Figure S1. IGF2BP1 RNA expression. **A** Distribution of RNA expression derived from bulk RNA-seq data of healthy tissues and cultured cells provided by the Human protein atlas (www.proteinatlas.org). **B** Distribution of RNA expression derived from bulk tumor RNA-seq data of the TCGA project, provided by GEPIA2 (http://gepia2.cancer-pku.cn). **C** Average RNA expression derived from single cell RNA-seq data of of healthy tissues aggregated by cell type provided by the Human protein atlas.

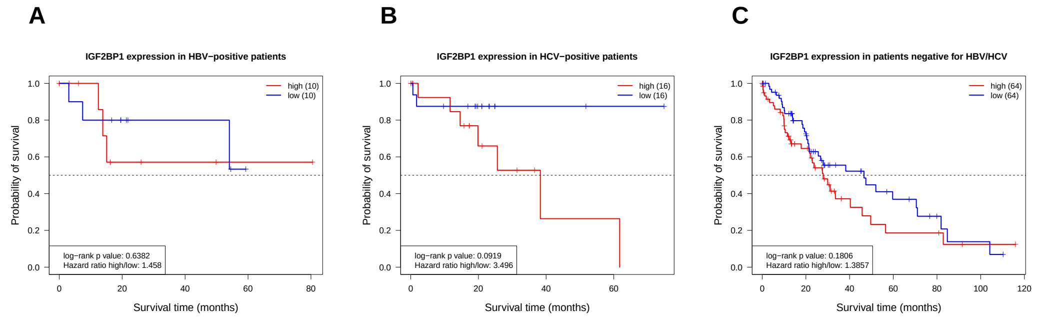

Figure S2. Kaplan-Meier curves describing overall survival probabilities depending on IGF2BP1 RNA expression in hepatocarcinoma derived RNA-seq data, provided by the TCGA (cohort LIHC). **A** Patients positive for HBV but not HCV. **B** Patients positive for HCV but not HBV virus. **C** Patients neither positive for HBV nor HCV. Patients were separated by median IGF2BP1 RNA expression.

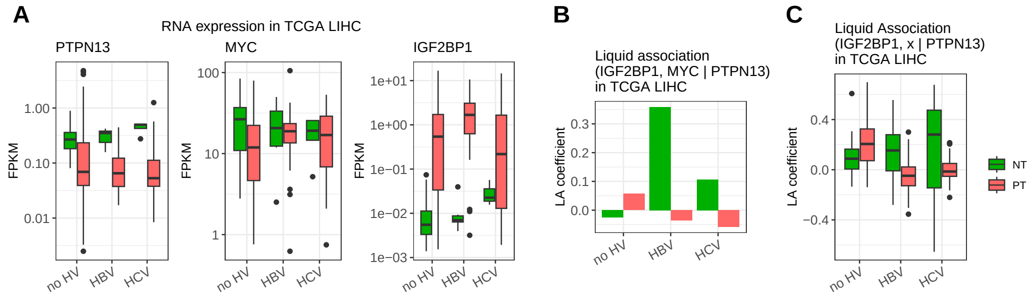

Figure S3. RNA expression and liquid association in in hepatocarcinoma derived RNA-seq data (HCC). **A** Distribution of RNA expression values of PTPN13, c-Myc and IGF2BP1 derived from HCC-RNA-seq samples (TCGA, cohort LIHC). **B** Liquid assocoation (LA) coefficients of the correlation between IGF2BP1 and c-Myc in dependence of PTPN13 in TCGA HCC samples. **C** LA coefficient distributions of the correlation between IGF2BP1 and 117 published target candidates in dependence of PTPN13 in TCGA HCC samples. no HV - samples without detected Hepatits virus infection, HBV - samples with exclusively detected Hepatitis B infection, HCV - samples with exclusively detected Hepatitis C infection, NT - normal tissue, PT - primary tumor tissue

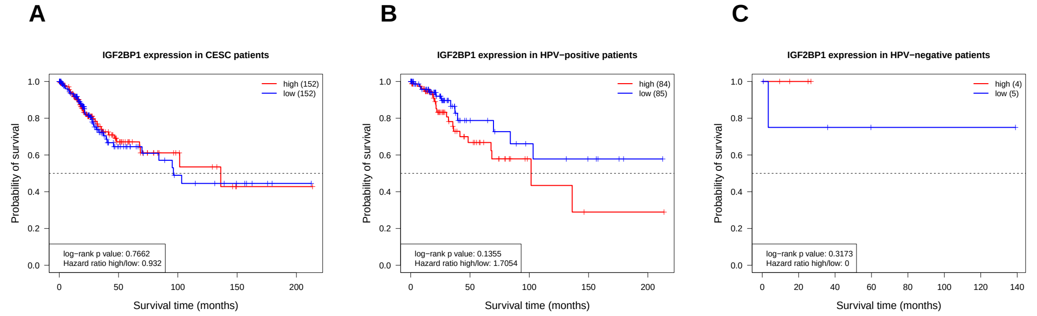

Figure S4. Kaplan-Meier curves describing overall survival probabilities depending on IGF2BP1 RNA expression in cervical carcinoma derived RNA-seq data, provided by the TCGA (cohort CESC). **A** Patients regardless of virus status. **B** Patients positive for HPV **C** Patients negative for HPV. Patients were separated by median IGF2BP1 RNA expression.

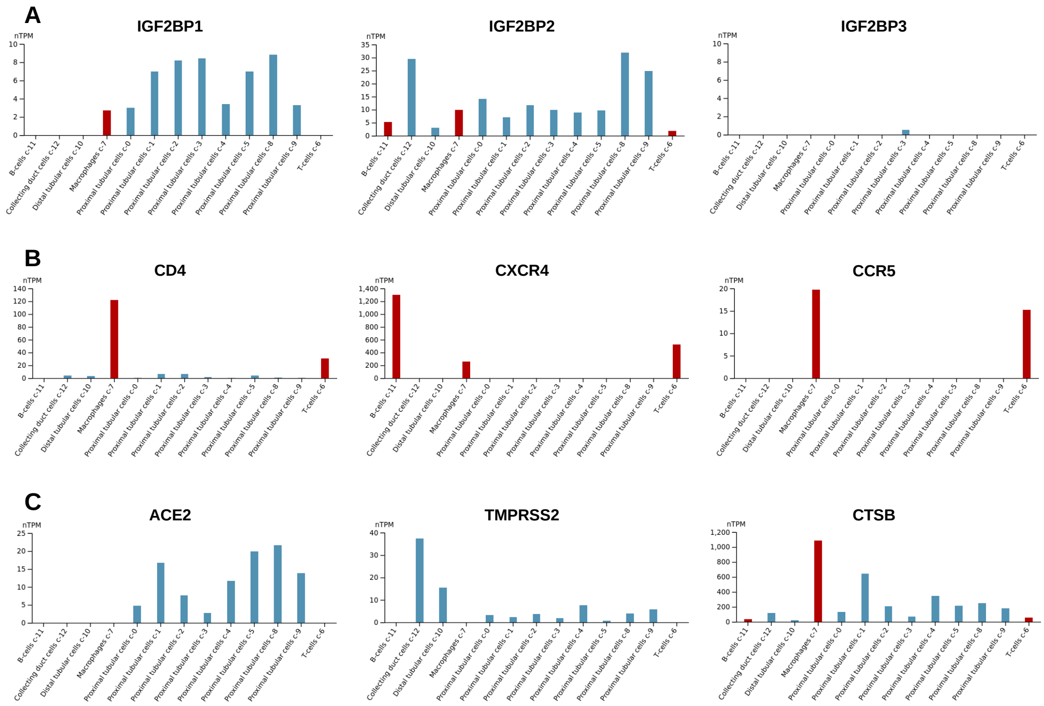

Figure S5. Average RNA expression in distinct kidney resided cell types derived from single cell RNA-seq data provided by the Human protein atlas (www.proteinatlas.org). **A** IGF2BPs. **B** HIV-1 host (co-)receptors. **C** SARS-CoV-2 host receptor and priming proteases
